# Expression profile of synaptic vesicle glycoprotein 2A, B, and C paralogues in temporal neocortex tissue from patients with temporal lobe epilepsy (TLE)

**DOI:** 10.1186/s13041-022-00931-w

**Published:** 2022-05-16

**Authors:** Burcu A. Pazarlar, Sanjay S. Aripaka, Viktor Petukhov, Lars Pinborg, Konstantin Khodosevich, Jens D. Mikkelsen

**Affiliations:** 1 Neurobiology Research Unit 8057, Neurological Clinic, Copenhagen University Hospital, Rigshospitalet, 6-8 Inge Lehmanns Vej, 2100 Copenhagen, Denmark; 2 Physiology Department, Faculty of Medicine, Izmir Katip Celebi University, Izmir, Turkey; 3 Biotech Research and Innovation Centre, Faculty of Health and Medical Sciences, University of Copenhagen, Copenhagen, Denmark; 4 Department of Neuroscience, University of Copenhagen, Copenhagen, Denmark; 5 Department of Neurology, Epilepsy Clinic, Copenhagen University Hospital, Rigshospitalet, Copenhagen, Denmark

**Keywords:** SV2 paralogues, Temporal lobe epilepsy, Neocortex, snRNA-seq, [^3^H]-UCB-J

## Abstract

Synaptic vesicle glycoprotein-2 (SV2) is a family of proteins consisting of SV2A, SV2B, and SV2C. This protein family has attracted attention in recent years after SV2A was shown to be an epileptic drug target and a perhaps a biomarker of synaptic density. So far, the anatomical localization of these proteins in the rodent and human brain have been reported, but co-expression of SV2 genes on a cellular level, their expressions in the human brain, comparison to radioligand binding, any possible regulation in epilepsy are not known. We have here analyzed the expression of SV2 genes in neuronal subtypes in the temporal neocortex in selected specimens by using single nucleus-RNA sequencing, and performed quantitative PCR in populations of temporal lobe epilepsy (TLE) patients and healthy controls. [^3^H]-UCB-J autoradiography was performed to analyze the correlation between the mRNA transcript and binding capacity to SV2A. Our data showed that the SV2A transcript is expressed in all glutamatergic and GABAergic cortical subtypes, while SV2B expression is restricted to only the glutamatergic neurons and SV2C has very limited expression in a small subgroup of GABAergic interneurons. The level of [^3^H]-UCB-J binding and the concentration of SV2A mRNA is strongly correlated in each patient, and the expression is lower in the TLE patients. There is no relationship between SV2A expression and age, sex, seizure frequency, duration of epilepsy, or whether patients were recently treated with levetiracetam or not. Collectively, these findings point out a neuronal subtype-specific distribution of the expression of the three SV2 genes, and the lower levels of both radioligand binding and expression further emphasize the significance of these proteins in this disease.

## Introduction

The synaptic vesicle glycoprotein 2 (SV2) family consists of three members which have 60% sequence homology encoded by three different genes, namely SV2A (chromosome 1), SV2B (chromosome 15), and, SV2C (chromosome 5) [1, 2]. SV2 proteins are mainly localized in integral membrane of pre-synaptic vesicles [4] and considered to play a crucial role in synaptic function [2–5].

Among these paralogues, SV2A has gained most interest as a target for the antiepileptic drugs levetiracetam, brivaracetam and seletracetam [6, 7]. SV2A selective radioligands have been used for positron emission tomography (PET) to determine the level of binding in psychiatric and neurological diseases as a proxy marker for synaptic density [8–14]. Changes in SV2A binding may not predict changes in synapses, but rather changes binding properties at the binding site in the SV2A protein. It is important to investigate if changes in binding reflects the same changes in gene expression and we therefore compared level of binding and gene expression in the same human tissues. Furthermore, we determined the phenotype of the cortical neurons to which the ligand bind, and any co-expression of the other two SV2 paralogues in the same cells.

It has been shown that SV2 proteins have a distinct distribution in the adult brain using immunohistochemistry and immunoblotting [2, 4, 15]. SV2A has the most ubiquitous expression and is considered expressed in neurons, and pharmacological evidence suggests presence in excitatory and inhibitory cortical synapses [16]. SV2B displays a more limited pattern than SV2A and is mainly expressed in the cerebellum, hippocampus, and basal ganglia [1, 16]. Lastly, SV2C shows an even more restricted localization in the basal ganglia and has been detected in low amounts in the neocortex [17–19].

However, based on the above background; little is known about broad range distributions of SV2 paralogues among principal neurons of the cortical layer and subtypes of interneurons in humans. The complexity of excitatory and inhibitor subtypes of cortical neurons is the backbone behind their local connectivity and functions [20–22]. This complexity urged us to elucidate the SV2 expression patterns across different subtypes of cortical neurons. Because we propose that distinct expressions of paralogues may reflect the diversity of cortical neurons and their differential involvement in epilepsy pathology.

It has been well documented that mRNA level, protein expression, and specific radioligand binding density of SV2A are markedly decreased in the hippocampus and the anterior temporal neocortex of temporal lobe epilepsy (TLE) patients as revealed by real-time PCR, immunohistochemistry, western blot, and neuroimaging studies [14, 23–26]. Another important aim of this investigation was to compare expression and binding, because this would increase our understanding of changes in binding capacity in imaging. Further, we compared the expression of the three paralogues to further investigate any co-regulation in the human brain.

With this report, we intended to use both temporal neocortex tissue resected from TLE patients and postmortem neocortical tissue from non-epileptic subjects and demonstrate shared or cell type-specific SV2 paralogue expression among subtypes of cortical neurons by the help of single nucleus RNA-sequencing (snRNA-seq). Another aim of this study is to investigate if any changes in expression correlate to our clinical data such as duration of epilepsy, seizure frequency, age, sex, and type of antiepileptic drug treatment during surgery.

## Materials and methods

### Patient information and tissue collection

Human neocortex samples were obtained from drug-resistant temporal lobe epilepsy patients undergoing temporal lobe resection surgery at the Department of Neurology and Neurosurgery at Rigshospitalet, Copenhagen. The temporal neocortex tissue is removed to reach the epileptogenic zone beneath it. The study was approved by the Ethical Committee in the Capital Region of Denmark (H-2-2011-104) and written informed consent was obtained from all patients before surgery. Non-epileptic postmortem brain materials were collected by the Human Brain Tissue Bank-Semmelweis University with approval of the Regional Committee of Science and Research Ethics of Semmelweis University. Permission numbers are No. 6008/8/2002 and No. 32/1992/TUKEB.

The tissues for the present experiments were collected from 22 TLE patients (13 females and 9 males, age between 18 and 58 years). All patients had focal impaired awareness seizures with variable frequency (1–12 seizures/month) during their epilepsy symptoms duration (4–44.5 years). According to magnetic resonance (MR) imaging results, all patients except 3 showed medial temporal sclerosis. The MR observations were confirmed by histopathological examination of the hippocampus in paraffin sections in all but one patients. Importantly, the cortical tissue was normal based on histopathological examination in all patients, but 2 had signs of focal cortical dysplasia. For all patients, there were detected EEG abnormalities in terms of ictal or interictal activities in involving the resected area (both hippocampus and neocortex). All patients were treatment resistant, but current or recent drug treatments as well as the time period from last levetiracetam dose in some of the patients were noted.

Postmortem brains were removed from the skull and snap-frozen within 1–6 h after death which is a very short postmortem delay, whereas postoperative brains were frozen within less than 30 min. Subsequently, all tissue samples were stored at − 80 °C. The concentration and quality of the were determined before further analysis.

### Single nucleus RNA sequencing data analysis

To determine the expression of the three SV2 genes in individual cell types of the human temporal cortex, we used epileptic temporal cortices originating from 9 TLE patients (out of the total of 22 patients in this study) and postmortem cortical tissues from 10 healthy controls. A detailed description of the procedures, as well as the relatively generated data, was recently created and published [27]. In brief and relevant for the present study, neuronal nuclei from the epileptic temporal cortex were isolated from the whole dataset, resulting in 117,221 single nucleus transcriptomes. Classification of neuronal clusters into subtypes of principal neurons and GABAergic interneurons was done as described previously [27]. The level of gene expression was estimated using log_10_-normalized transcript per million mapped reads.

### Gene expression with quantitative polymerase chain reaction (qPCR) and data analysis

Total RNA was extracted using Quick-RNA MiniPrep Kit (ZymoResearch, R10564) from temporal cortex resections of 22 TLE patients. The tissue was collected as tissue sections, and all layers of the cortex were represented in the samples. RNA concentration was determined by Nanodrop microvolume spectrophotometer (Thermofisher, 2000/2000c UV–Vis) and purified total RNA was reverse transcripted to single-stranded cDNA by using Promega reverse transcription system (A3800) and the BioRad ICycler Thermal Cycler Instrument. cDNA was synthesized in a final volume of 20 µL reaction system containing 4 µL Improm-11 5× Reaction Buffer, 4.8 µL MgCl_2_, 1 µL dNTP mix, 0.5 µL Recombinant Rnasin Ribonuclease Inhibitor, 1 µL oligo(dT) and 7.7 µL RNA. As a first step, 1 µL oligo(dT) and 7.7 µL RNA sample mixed and incubated for 5 min at 60 °C in an iCycler (Bio-Rad), in order to amplify mRNA specifically and accelerate the mRNA’s poly-A tale recognition. The following conditions; 5 min at 25 °C, 60 min at 42 °C, 15 min at 72 °C were used in the second step of reverse transcription.

qPCR of reverse-transcribed samples (qRT-PCR) was performed using Bio-Rad IQ SYBR-Green Supermix (170-8880) and Roche Light Cycler 480 II. The reaction was carried out in a final volume of 20 µL containing 11 µL IQ SYBR green super mix (Bio-Rad), 5 µL cDNA, 15 pmol Forward Primer, 15 pmol Reverse Primer, and RNase free water. The following conditions, 3 min at 95 °C (1 cycle), 15 s at 95 °C, 30 s at 60 °C, 30 s at 72 °C (40 cycles) were used for reverse transcription PCR. The specificity of the products was assessed using a melting curve analysis.

Primer design was performed using NCBI BLAST tool and Primer3web tool. The sequence information for all primers used in the qRT-PCR analysis is provided in Table 1. The level of expression was determined by absolute quantification of three paralogues and one internal control gene GAPDH. For absolute quantification, the known concentration of template used to construct the standard curve and expression levels of the gene of interests in the unknown sample was determined by interpolation method. Expression levels of SV2 paralogues were then normalized by dividing the level of the gene of interests by the level of housekeeping gene GAPDH in the same sample. The ratio of SV2 paralogue/GAPDH mRNA level (mean ± standard deviation) was computed in the analysis.

**Table 1:** Primer information

Gene	Gene ID	Sequence	Length (bp)	Product length (bp)	TM
SV2A human	NM_014849.4	F: 5′-AGCGTGATGTCCTGTGTCTC-3′	20	168	59.97
		R: 5′-GCCAAAAGCTGTGGTCCTCTT-3′	21		59.96
SV2B human	NM_001167580.2	F: 5′-TGCCCTGTACTGTGTGATGG	20	70	59.68
		R: 5′-CATGGCAAACCAAACCACGG	20		60.60
SV2C HUMAN	NM_014979.3	F: 5′-ATGATCGGTGGCATCTACGC-3′	20	117	63.3
		R: 5′-GACGATGACAAACACACGCC-3′	20		62.6
GAPDH human	NM_002046.7	F: 5′-CATGAGAAGTATGACAACAGCCT-3′	23	113	58.49
		R: 5′-AGTCCTTCCACGATACCAAAGT-3′	22		59.10

### Autoradiography

Twelve µm-thick coronal sections from patients and postmortem cortical tissue were mounted on super frost pre-gelatinized glass slides (Thermo scientific). Brain sections were pre-incubated twice for 10 min at room temperature in a 50 mM Tris–HCl buffer (pH 7.4) with 0.5% bovine serum albumin (BSA). Incubation was performed for 60 min in a 50 mM Tris–HCl buffer (pH 7.4) containing 0.5% BSA, 5 mM MgCl_2_, 2 mM EGTA, and 3 nM [^3^H]-UCB-J (UCB Pharma, Belgium). Subsequently, slides were washed twice in an ice-cold pre-incubation buffer for 10 min and briefly dipped in ice-cold distilled water. Glass slides were then kept in a paraformaldehyde chamber and exposed to FUJI imaging phosphor plates for 3 days at 4 °C together with [^3^H] standard ARC (American Radiolabeled Chemicals, Inc, USA) and [^3^H] microscale Batch 21A (GE Healthcare, UK). The FUJI imaging plates were scanned by Fujifilm Image Reader (BAS-2500 V1.8) and autoradiograms were analyzed with Image J software (Version 2.0.0, NIH). Non-specific binding was determined by adding 10 mM levetiracetam in the incubation solution. The grey value optical densities were correlated to the standards of known concentrations [nCi/mg]. The interpolated values were then calculated into the amount of bound radioligand [fmol/mg TE] in the tissue.

### Statistical analysis

All statistical analyses were performed in GraphPad Prism (v 8.2.0), or R (v 3.6.0). The number of patients and the related statistical information were stated in the corresponding figure legends. The distribution of dependent variables was assessed using the D'Agostino & Pearson normality test. The statistical analysis of single cell data was conducted as earlier described in details [27]. For analysis of the correlation between different paralogues nonparametric Spearman’s correlation test was performed as the data set was not normally distributed. Mann Whitney U test (nonparametric) was applied for comparison of expression between sex groups and between LEV versus other AED treated patients since data were not distributed normally. Spearman’s correlation analysis was performed for analysis of the correlation between expression levels and age, duration of epilepsy, seizure frequency since data sets were not distributed normally. To compare the [^3^H]-UCB-J binding level between postmortem tissue from non-epileptic controls versus resected tissue from temporal lobe epilepsy patients, parametric unpaired Student’s t-test was carried out for data set was passed normality test. Parametric Pearson’s correlation analysis was performed to analyze the correlation between transcript level and [^3^H]-UCB-J binding level as the data set was distributed normally.

## Results

### Distinct expression patterns of SV2 genes among subtypes of neocortical neurons in normal subjects and TLE patients

**Fig. 1:**
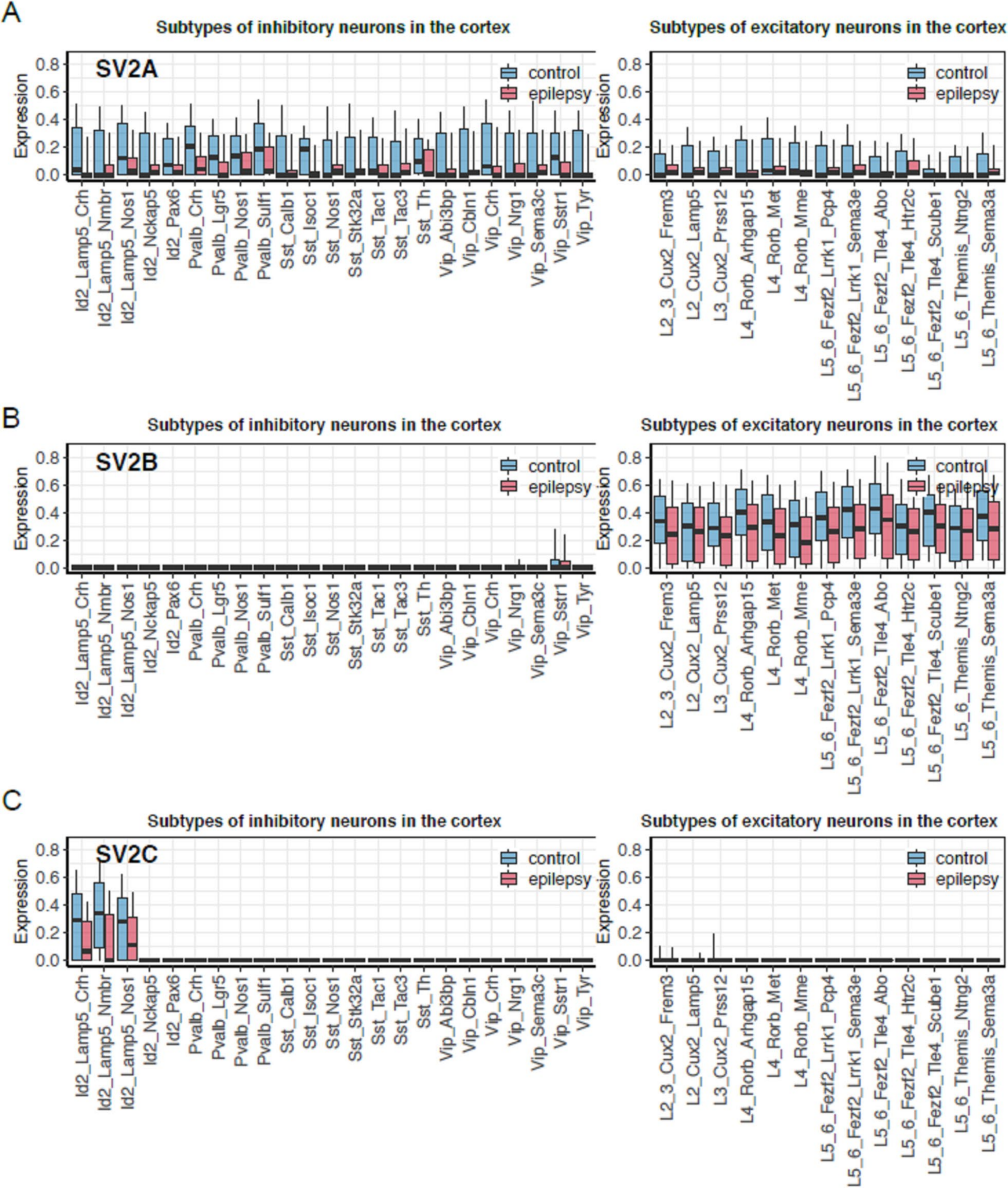
Levels of SV2A (**A**), SV2B (**B**) and, SV2C (**C**) among cardinal subtypes of cortical neurons. Data presents expression levels of SV2A (**A**), SV2B (**B**) and, SV2C (**C**) transcripts among cardinal subtypes of GABAergic and Glutamatergic neurons in temporal neocortex of TLE (temporal lobe epilepsy) patients (n = 9) and non-epileptic postmortem controls (n = 10) by single nucleus RNA sequencing. Distribution of log-normalized expression (y-axis) per cell types (x-axis) is shown for SV2A (**A**), SV2B (**B**) and SV2C (**C**). First, 10%, 25%, 50%, 75% and 90% of expression were estimated for each patient and cell type. Then, trimmed means of these values across samples were estimated for each condition and cell type (trim = 0.3). These averaged values are shown with boxplots: the middle line represents median, lower and upper hinges correspond to quartiles (25% and 75%) and whiskers represent 10% and 90% of expression. When there are no whiskers present, the expression is 0 across all cells of a subtype. Left panels indicates Parvalbumin (Pvalb), Somatostatin (Sst), Inhibitor of DNA Binding-2 (Id-2), Vasoactive Intestinal Polypeptide (Vip) expressing neurons which are the main four cardinal classes of GABAergic neurons. Excitatory glutamatergic neurons of five principal layers in the cortex (i.e. L2, L3, L4, L5, and L6) were shown in the right panels (The single cell data are derived from Pfisterer et al. [27])

The single-cell analysis in the temporal cortex is based on 9 TLE patients and 10 matched non-epileptic postmortem controls. First, we identified the distribution of three different SV2 mRNA expressions among neuronal subtypes of neocortical tissues of non-epileptic postmortem controls. In these cortical tissues, we found substantial expression of SV2A level in all subtypes of GABAergic (Fig. 1A, Right panel) and glutamatergic neurons (Fig. 1A, Left panel) of the neocortex. The SV2A level was overall slightly higher in GABAergic subtypes compared to glutamatergic subtypes. We also revealed prominent expression of SV2B that was specific for principal glutamatergic neurons for all cortical layers (Fig. 1B, Left panel). In contrast, the expression of SV2C was negligible in glutamatergic subtypes (Fig. 1C, Left panel) whereas it had a highly-specific expression that was limited to the inhibitor of DNA binding-2 family of GABAergic interneurons that mainly reside in L1-3 (Fig. 1C, Right panel).

In the temporal neocortex of epileptic patients, we found that the level of SV2A expression was lower in all subtypes of both GABAergic interneurons and glutamatergic principal neurons (Fig. 1A). Similarly, the expression of SV2B in principal neurons (Fig. 1B, Right panel) and SV2C level in the subset of interneurons was also shown to be lower in patients (Fig. 1C, Right panel). Due to variability of the data, the relatively few samples included, and the multiple comparisons, there are no overall statistical differences between TLE patients and postmortem controls.

### A positive correlation was observed between SV2A and SV2B in the temporal neocortex of TLE patients

**Fig. 2:**
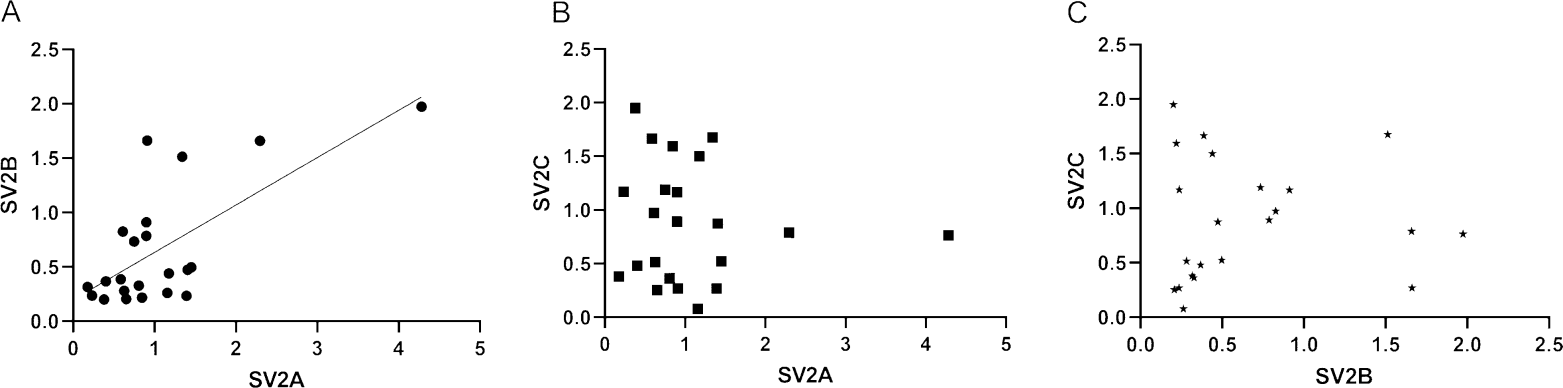
Inter-paralogue correlations between SV2 mRNA levels in the temporal cortex of TLE patients. SV2A showed a significant positive correlation with SV2B (ρ = 0.5189, *p* = 0.0133, 95% confidence interval) (**A**), but SV2A is not correlated with SV2C (ρ = − 0.1462, *p* = 0.51) (**B**). There is also no correlation between SV2B and SV2C (ρ = 0.1158*, p* = 0.60) (**C**). p < 0.05 is considered statistically significant for all comparisons. ρ (rho) represents the Spearmen’s correlation coefficient (n = 22). Investigated correlations were linear

We further compared the relative expression of SV2A and SV2B levels in the temporal cortex from 22 TLE patients; the expression of the SV2A and SV2B genes were significantly correlated (ρ = 0.5189, *p* = 0.013, 95% confidence interval) (Fig. 2A). As the SV2B is expressed only in the glutamatergic neurons, this type of neuron would be the cellular substrate for co-expression of SV2A and SV2B. By contrast, no correlation was found between SV2C mRNA levels and the other two SV2 transcripts (Fig. 2B, C).

### Neither sex nor age influences the expression levels of SV2 paralogues in TLE patients

The analysis using the Mann–Whitney U test revealed no difference in expression between sexes. [SV2A (*p* = 0.97, U = 55), SV2B (*p* = 0.36, U = 42) and SV2C levels (*p* = 0.66, U = 49)]. Further, there were no evidence that either SV2A (ρ = − 0.1125, *p* = 0.61), SV2B (ρ = − 0.002, *p* = 0.99) or SV2C (ρ = 0.083, *p* = 0.71) expression were correlated with age among the involved patients.

### Duration of epilepsy and frequency of seizures could not be related SV2 genes expression

Spearman’s correlation coefficient showed there were no correlation between duration of epilepsy (years from onset) and SV2 genes expression [SV2A (ρ = 0.1283, *p* = 0.56), SV2B (ρ = 0.217, *p* = 0.33) or SV2C (ρ = − 0.154, *p* = 0.79)].

The seizure pattern was the complex focal seizure with different seizure frequencies for all patients included. Seizure frequency was also shown not related to SV2 gene expression [SV2A (ρ = 0.318, *p* = 0.14), SV2B (ρ = 0.039, *p* = 0.86) or SV2C (ρ = − 0.034, *p* = 0.88)].

### There was no change in SV2A gene expression level in patients treated with levetiracetam

According to Mann–Whitney-U test results, there was no statistical difference in SV2A (*p* = 0.88, U = 56), SV2B (*p* = 0.35, U = 44), and SV2C (*p* = 0.75, U = 57) expression level when we compared the levetiracetam treated patients (n = 13) and other anti-epileptic drug (n = 9) treated patients before surgery.

### [^3^H]-UCB-J binding which reflects SV2A level shows a positive correlation with SV2A transcript level

**Fig. 3:**
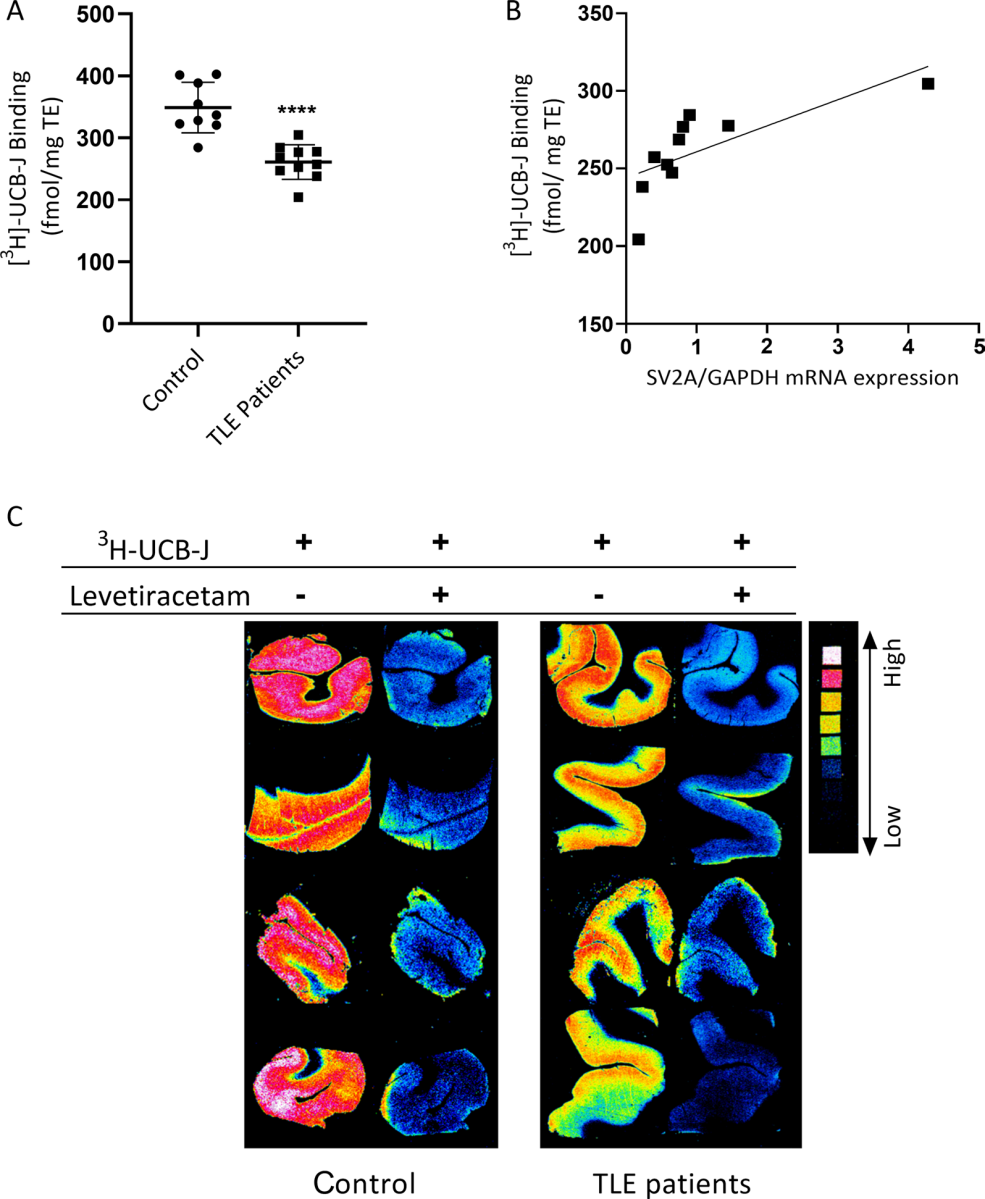
[^3^H]-UCB-J binding level in cortex tissue and correlation of SV2A mRNA level with [^3^H]-UCB-J binding. Quantitative autoradiographic measurements of cortical [^3^H]-UCB-J density is expressed as specific binding in nCi/mg tissue in cortex of TLE (temporal lobe epilepsy) patients (n = 10) and non-epileptic postmortem controls (n = 9) (Student’s *t*-test*; p* < *0.001*, t = 5.506, df = 17, 95% confidence interval). All the values are in Mean ± SD (**A**). Positive correlation between SV2A gene expression levels and [^3^H]-UCB-J binding levels of patients (n = 10, r = 0.72, *p* = *0.018*, 95% confidence interval). r represents the Pearson’s correlation coefficient. p < 0.05 is considered statistically significant for all comparisons. Investigated correlations were linear. **B** Four different representative autoradiogram images of cortex from both TLE patients and controls. Non-specific binding was shown with 10 mM levetiracetam addition to incubation solution of adjacent slice (**C**)

Using neocortical tissue sections from 9 TLE patients and 10 postmortem non-epileptic controls, we measured the binding level of [^3^H]-UCB-J using *in-vitro* autoradiography. According to unpaired Student’s *t*-test comparison [^3^H]-UCB-J binding was significantly lower in TLE patients when compared to controls (*p* < 0.001, t = 5.506, df = 17, 95% confidence interval) (Fig. 3A, C). Importantly, there was a significant positive correlation between SV2A gene expression levels and [^3^H]-UCB-J binding levels of patients according to Pearson test (r = 0.72, *p* = 0.018) (Fig. 3B).

## Discussion

Studies conducted in rodents have contributed to the understanding of the anatomical distribution of SV2 proteins by immunohistochemistry, in situ hybridization, and western blot techniques [17, 28–31]. It is also generally believed that SV2A is distributed throughout all brain areas in humans as evidenced by in-vitro autoradiography mapping [32], and SV2C is located in the human substantia nigra in humans similar to rodents [18]. The present study supports these observations and extends it to all family members. Further, at least for the human temporal cortex the SV2A transcript is present in all neurons supporting the wide distribution in rodents using immunodetection and binding studies in human [9, 32, 45].

A limited number of studies have measured SV2A levels in the human neocortex with intractable epilepsy and cortical dysplasia [24, 25, 33]. Little is also known about broad range distribution of paralogues and their shared expression in the neocortex tissue. In this regard, our data provide novel knowledge about expression patterns of SV2 paralogues in the temporal neocortex both in healthy humans as well as in patients with epilepsy.

Neocortical neurons are originally divided in GABAergic interneurons [34] and glutamatergic pyramidal neurons [35], but these are further divided in subtypes based on their co-expression of other markers and their functional properties [34–36]. Single nucleotide RNA-seq methods provide a good opportunity to discriminate and identify different transcripts in various cell populations in heterogeneous tissues such as the cerebral cortex [37]. In this context, using snRNA-seq dataset from the resected cortex of TLE patients and postmortem cortical tissue of normal subjects, we identified expression of SV2 paralogues among subclasses of GABAergic inhibitory interneurons of cortex such as parvalbumin, somatostatin, Inhibitor of DNA binding-2, vasoactive intestinal polypeptide expressing neurons and excitatory glutamatergic neurons of all five principal cellular layers in the cortex.

SV2B is expressed in all glutamatergic neurons of principal layers, but not in the inhibitory neurons. These results are in line with the evidence from quantitative co-localization studies of SV2 proteins and vesicular transporters of GABA and glutamate [2, 5, 28]. Earlier immunocytochemical studies conducted in cultured cortical neurons showed that SV2A is expressed in GABAergic neurons, while SV2B is predominately expressed in glutamatergic neurons [28, 29, 38]. Additionally, our finding shows SV2C is located in only one group of GABAergic interneurons of the cortex as well as in other inhibitory neurons in the striatum and cerebellum in both rodents and humans [17, 18].

Mosaicism in the cytoarchitecture has a substantial impact on controlling the excitation/inhibition balance of cortical networks [39, 40]. Although all GABAergic cortical neurons are inhibitory their functional properties in local circuits are different and dependent on their content of other neurotransmitters [40–42]. Considering epilepsy as a cortical neuronal network disease [43], the SV2 heterogeneity among excitatory and inhibitory neurons could be important. Our results support the idea that heterogeneity in SV2 paralogue distribution among subtypes of cortical neurons determines possible paralogue-dependent function.

Although the investigated cortices are not the seizure initiating zone, we could not exclude the occurrence of abnormal EEG in this region. Different vulnerabilities of different regions with or without epileptic activity should be further clarified. Importantly, Groot et al. [44] showed no significant differences in the SV2A immunoreactivity in the peritumoral cortex of the patients with or without epileptic activity.

Further, we showed that the neocortical expression pattern of SV2A and SV2B is positively correlated. In contrast, SV2C is not co-expressed with any of the other paralogues. Notably, SV2A/SV2B double knock-out mice show more severe pathological outcomes when compare to mice with only SV2A mutation [1] suggesting that the two gene products share functions. For the human cortex, SV2A and SV2B are co-expressed in excitatory cortical neurons, but may not overlap in all brain regions [1, 16].

We would anticipate that SV2A and SV2B are also co-expressed in the hippocampal excitatory neurons, and the loss of each and both in genetically modified mice, may drive their phenotypes towards convusions. The reduced level of SV2A expression in the hippocampus correlating to the hippocampal sclerosis degree [25] supports this hypothesis.

It can be speculated that there is a broad range of expression levels of SV2 paralogues among subjects consistent with variabilities in terms of age, sex, treatments, and duration of epilepsy. Our results showed no relationship with sex in accordance with a recent PET study [45]. SV2A protein expression has been reported to increase postnatally in the rat brain [46–48]. A recent PET study showed no differences in SV2A binding during adult aging in healthy subjects [45], and our data agree that no change occurs under adult ageing in TLE patients.

In the relatively few patients included here, there were no differences in SV2 paralogues expression between levetiracetam- and other antiepileptic drugs-treated patients. It is well known that levetiracetam exerts an anticonvulsant effect by binding SV2A [4], while other antiepileptic drugs reduce the excitability of the brain by modulating other targets [49]. Because patients in treatment with levetiracetam or brivaracetam had no difference in binding of the radiotracer when compared to other patients we suggest that the binding site is not occupied to a major extent by these drugs and they have likely no effect on SV2A expression.

Different seizure patterns may also impact on SV2 levels. However, seizure frequency/month was not found to correlate to SV2 expression.

Although it is proposed that SV2A mRNA and protein levels showed a positive correlation in brain structures of rats [47], these correlations have not been performed in the human brain and not for binding. We show here that SV2A gene expression is strongly correlated with [^3^H]-UCB-J binding as another tool of measuring SV2A levels. In agreement with prior studies [14], the bound [^3^H]-UCB-J in grey areas was 25% lower in resected cortical tissue of TLE patients compared to postmortem cortical tissue of controls. The PCR data support the qualitative single cell data, revealing a reduction of SV2A expression in the patients.

Previously, decreased SV2A binding has been reported only in the hippocampus and amygdala, probably due to sclerosis pathology in medically refractory TLE patients [14, 26]. Moreover, reduced SV2A level in relation to sclerosis degree in the hippocampus has previously been demonstrated both in epileptic rats [23] and TLE subjects [25]. Although previous studies showed that the reduction in SV2A is mostly localized in the medial temporal lobe, we now also report that SV2A is reduced in the lateral temporal cortex as well.

## Conclusion

This translational report contributes to the understanding of shared and cell-type-specific transcript expressions of SV2A, SV2B, and SV2C among different inhibitory and excitatory neuronal subtypes in the neocortex in both health and disease. Results of this study provide insight into the link between basic clinical outcomes and SV2A expression**.**

## Data Availability

We confirm that all the work described in the manuscript is consistent with Journal’s guidelines for ethical publication. All data generated or analysed during this study are available from corresponding author on reasonable request.
